# Comparative study on the clinical application of mixed reality technology leading micro-invasive intervertebral foramen puncture location and blind puncture location

**DOI:** 10.12669/pjms.36.3.1683

**Published:** 2020

**Authors:** Ma-long Guo, Song-tao Yue, Jiang-yi Wang, Hong-xun Cui

**Affiliations:** 1 Ma-long Guo, Department of Orthopedics, Orthopedic Hospital of Henan Province, Luoyang, Henan 471000, P.R. China; 2 Song-tao Yue, Department of Orthopedics, Orthopedic Hospital of Henan Province, Luoyang, Henan 471000, P.R. China; 3 Jiang-yi Wang, Department of Orthopedics, Orthopedic Hospital of Henan Province, Luoyang, Henan 471000, P.R. China; 4 Hong-xun Cui, Department of Orthopedics, Orthopedic Hospital of Henan Province, Luoyang, Henan 471000, P.R. China

**Keywords:** Intervertebral disc herniation, Intervertebral foramen microscope, MR technology, Puncture locating

## Abstract

**Objective:**

To discuss the function of mixed reality (MR) technology in guiding location of intervertebral foramen microscopic puncture and analyze its feasibility and clinical application value.

**Methods:**

Sixty patients with lumbar intervertebral disc who were treated between January 2017 to October 2017 were chosen, and classified into navigation group (30 cases) and traditional control (30 cases) according to random number table. Intervertebral foramen microscopic operation was conducted for both groups. MR technology was applied for the navigation group to guide puncture and establish intervertebral foramen microscopic cannula. Traditional C-arm X-ray apparatus was used for traditional group to establish intervertebral foramen microscopic cannula. Intra-operative puncture times, fluoroscopy times, puncture time and VAS score 1d, 3m and 6m after the operation were recorded and compared.

**Results:**

Postoperative waist and leg pain symptoms of both groups were relieved obviously, and straight leg raising test for the diseased limb turned to be negative. Intra-operative puncture times, fluoroscopy times, puncture time and operation time had statistical significance decrease.

**Conclusion:**

Mixed reality (MR) can accurately guide the establishment of intervertebral foramen microscopic cannula, solve the bottleneck problem of intervertebral foramen microscopic technology, promote puncture success rate, reduce repeated puncture times, avoid by-injury, shorten puncture time and reduce X-ray radiation quantity of operators and patients, so it deserves to be promoted and applied.

## INTRODUCTION

Operative treatment of lumbar intervertebral disc herniation has gained the favorable clinical effect. Compared with traditional operation, intervertebral foramen microscopic technology punctures from the rear side of lumbar vertebra under local anaesthesia, which will neither damage the rear muscle nor destroy important arthrosis ligament structure of lumbar vertebra. Besides, it has no obvious influence on lumbar vertebra stability. It is unnecessary to separate and drag nerve root and dural sac, and there is no obvious disturbance of intraspinal nervous tissue. Meanwhile, it also has the advantages of small operative wound, a little hemorrhage, short time in bed, low operation cost and fast recovery.^1^ In recent years, this technology has been mastered by most doctors of minimally invasive spine surgery. Puncture locating guided by X-ray is the key to the operation. The traditional puncture method utilizes X-ray locating to gradually adjust puncture needle position and enter the target, and repeated fluoroscopy is required, which increases operation time and radiation exposure.^2^ Clinicians have been trying to reduce puncture times, decrease fluoroscopy times and improve operation safety/curative effect. MR technology drives the development of minimally invasive surgery.^3^ This study was done to assess the increased ease and accuracy of surgery when done by using both the methods. Thus, we utilized MR technology for locating. Thirty patients who received lumbar intervertebral disc excision by intervertebral foramen microscopic technology were studied and the good clinical effect was gained. They were compared with 30 patients for whom traditional C-arm X-ray apparatus was used to guide cannula establishment. The reason was to document the differences between both traditional or the MR technique.

## METHODS

Sixty patients with lumbar intervertebral disc who had been treated in our hospital from January 2017 to October 2017 were chosen. The study was approved by the Institutional Ethics Committee (Dated: 12 September 2019) of Orthopedic Hospital of Henan Province, and written informed consent was obtained from all participants .With the consent of Ethics Committees of the hospital, the subjects or their relatives agreed and signed the consent Form.

### Inclusion criteria

lumbar intervertebral disc herniation, including prolapse, dissociation and giant intervertebral disc herniation.

### Exclusion criteria

non-lumbar intervertebral disc herniation or waist and leg pain caused by degeneration; lumbar spondylolisthesis, lumbar spinal stenosis, over-high crista iliaca, lumbar intervertebral disc herniation and protrusion calcification. All patients were classified into navigation group (30 cases) and traditional control (30 cases) according to random number table, as shown in Table-I. The navigation group included 19 male patients and 11 female patients, with the age of 19-61 and average age of 40.2±5.3. All of them were in L5/S1 segment. There were 26 patients with side herniation and four patients with paracentral herniation. VAS score of preoperative leg pain was 5.3~9.6, with the average score of 8.1±2.8. The traditional group included 17 male patients and 13 female patients, with the age of 21-60 and average age of 41.4+4.9. All of them were in L5/S1 segment. There were 22 patients with side herniation and eight patients with paracentral herniation. VAS score of preoperative leg pain was 5.4~9.5, with the average score of 7.9±3.1. The differences of both groups in gender, age, herniation segment, herniation type and VAS score of preoperative leg pain had no statistical significance (p>0.05). Preoperative straight leg raising test of both groups was positive, and there was no combined and severe medical diseases. Lumbar vertebra MRI examination was carried out before the operation. Intervertebral foramen microscopic treatment was performed for both groups. The same operation group and the same doctor executed the operation. MR technology was used for the navigation group to establish intervertebral foramen microscopic cannula, while the traditional group adopted traditional C-arm x-ray apparatus to establish intervertebral foramen microscopic cannula.

**Table-I:** Patients’ basic information before the operation.

	Age	Male/Female	Preoperative VAS score	Operation segment
Navigation locating group	40.2±5.3	19/11	8.1±2.8	L5/S1
C-arm x-ray locating group	41.4+4.9	17/13	7.9±3.1	L5/S1
p	p>0.05	/	p>0.05	/

### Preoperative Preparation

Joimax’s intervertebral foramen microscopic work system (Joimax, Germany) was applied for both groups. The navigation group used MR system for puncture locating, while the traditional group adopted Joimax puncture needle for locating. 0.19mg phenobarbital sodium and 0.25mg atropine were intramuscularly injected for both groups before the operation. Local infiltration anesthesia and intravenous injection of dexmedetomidine were applied for anesthesia. 0.5 dexmedetomidine load dosage was given 10min before the operation started.

### Position and body surface marking

The patients took prone position and bent the hips properly to make intervertebral foramen stretch to the greatest extent. Spinous process ligature of lumbar vertebra, horizontal line of diseased intervertebral disc and puncture line were marked and located under C-arm X-ray apparatus. L3/4 puncture point was cut for 8~10cm from the side of middle line of spinous process. Similarly, L4/5 10-12cm and L5/S1 12~14cm. Puncture locating: for the navigation group, the navigation reference frame was installed and fixed on the spina iliaca posterior superior of uneffected side; navigation adapter was installed and fixed at the near end of guide rod; C-arm X-ray apparatus gathered anterioposterior and lateral views of lumbar vertebra. Under the guidance of computer navigation, the guide rod was used for puncture. The position of puncture target point was under the normal perspective. The puncture needle point was on the ligature of upper and lower pedicle of vertebral arch. Under lateral perspective, the puncture needle point was on the ligature of posteriors of upper and lower adjacent centrums. After arriving at the target point, the fine guide needle was imbedded. C-arm X-ray apparatus was used for anteroposterior perspective to confirm puncture position, and the guide rod was pulled out. Intervertebral foramen tools (Grade 1 and Grade 2 guide rods, catheter and trepan) were used to expand the tube level by level. Finally, the cannula with the diameter of 7.5mm was imbedded to connect intervertebral foramen microscope.

### Operation under microscope

According to the size and position of herniated lumbar intervertebral disc, nucleus pulposus pincers of different model and angle were used to take out herniated, prolapsed or dissociated nucleus pulposus tissues. According to the observation, the hard membrane beat well, and the nerve root was released. Through communication with the patients, their waist and leg pains were relieved. Intervertebral foramen microscope and cannula were removed. Then, the incision was sutured and bound up. After the patients in the traditional group were anesthetized, #18 puncture needle was inserted slowly. C-arm X-ray apparatus was used for anteroposterior and lateral X-ray perspective. It was verified that the puncture needle reached the target point position. The follow-up intervertebral foramen microscopic operation was same with the navigation group.

### Assessment Method

Puncture times, fluoroscopy times and puncture time of both groups in the operation process were recorded. Meanwhile, VAS scores of both groups before the operation, 1d after the operation, 3m after the operation and 6m after the operation were recorded. Puncture times, fluoroscopy times and puncture time as well as VAS scores of both groups 1d, 3m and 6m after the operation were compared.

### Statistical Method

SPSS 21.0 was used for statistical analysis (SPSS, US). Measurement data were expressed with μ±x. Independent-samples *T* test was applied. p<0.05 difference was taken as statistically significant.

## RESULTS

The navigation group in intraoperative puncture times, fluoroscopy times and puncture time had significance decrease than traditional control groups (p<0.05, Table-II). VAS scores 1d, 3m and 6m after the operation are shown in Table-III. VAS scores of both groups decreased, compared with pre-operation (p<0.05, Table-IV). The differences of both groups in VAS scores at the same time point had no statistical significance (p>0.05).

**Table-II:** The differences of both groups in intraoperative puncture times, fluoroscopy times and puncture time.

	Puncture times	Fluoroscopy times	Puncture locating time	Operation time
Navigation locating group	2.12±0.31	8.30±1.24	5.05±2.0	101.14±9.58
C-arm X-ray fluoroscopy locating group	10.53±5.55	60.00±15.15	34.80±6.32	129.72±14.36
p	p<0.05	p<0.05	p<0.05	<0.05

**Table-III:** Preoperative and postoperative VAS (Visual Analogue Score) scores.

	Postoperative	1d	3m	6m
Navigation locating group	8.1±2.8	2.6±0.8	1.8±0.4	1.4±0.3
C-arm X-ray fluoroscopy locating group	7.9±3.1	2.8±1.1	2.1±0.2	1.6±0.5
p	p>0.05	p>0.05	p>0.05	p>0.05

**Table-IV:** VAS scores of both groups decreased, compared with pre-operation.

Comparison of Groups	Postoperative versus preoperative	p
*Navigation locating group*		
1d versus preoperative	2.6±0.8 versus 8.1±2.8	p<0.05
3m versus preoperative	1.8±0.4 versus 8.1±2.8	p<0.05
6m versus preoperative	1.4±0.3 versus 8.1±2.8	p<0.05
*C-arm X-ray fluoroscopy locating group*		
1d versus preoperative	2.8±1.1 versus 7.9±3.1	p<0.05
3m versus preoperative	2.1±0.2 versus 7.9±3.1	p<0.05
6m versus preoperative	1.6±0.5 versus 7.9±3.1	p<0.05

**Fig.1:**
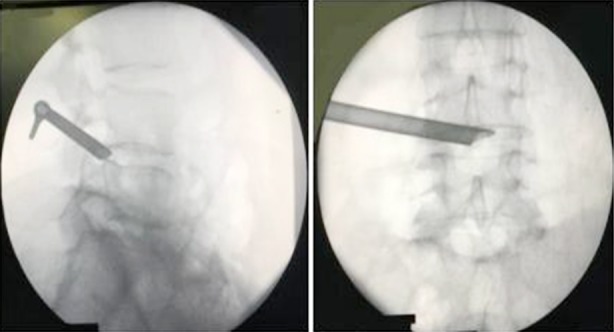
Navigation guided by mixed reality technology.

**Fig.2:**
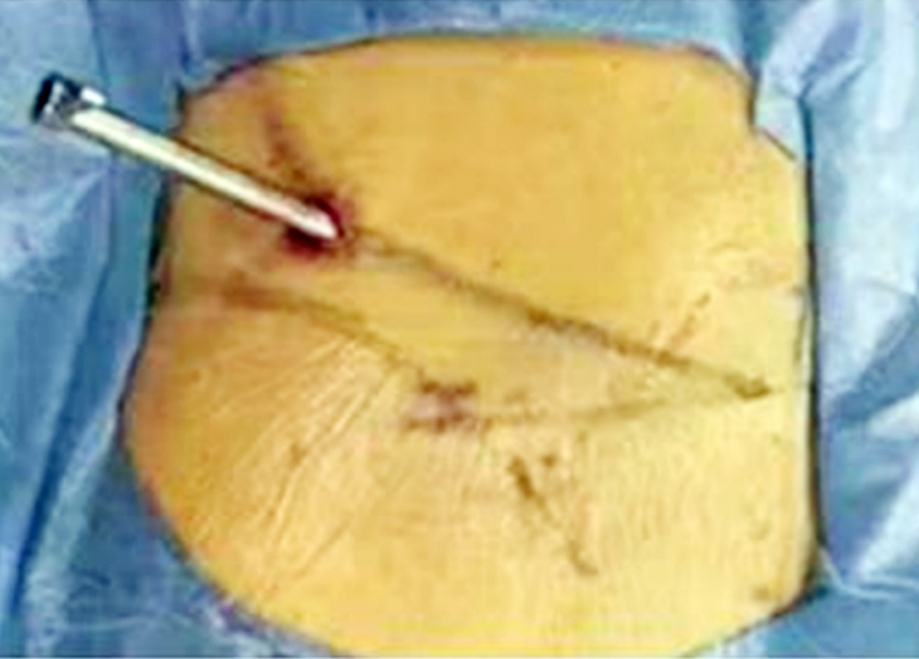
Through the mixed reality technology to guide the operation channel established by puncture.

**Fig.3:**
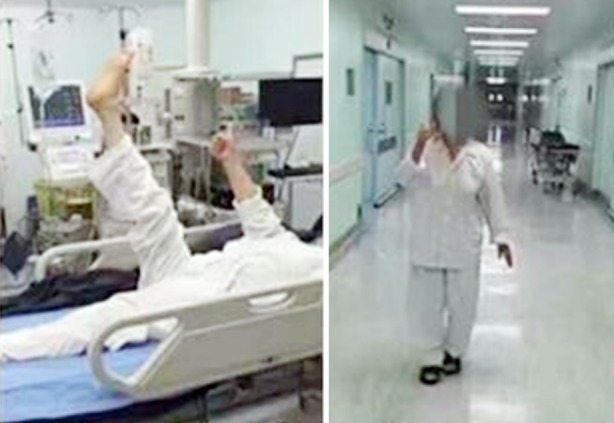
Immediately after the operation, the patient was out of bed and the pain disappeared.

In the navigation group, 21 patients were punctured in place within two needles, and only nine patients failed to achieve the puncture effect of being punctured in place within two needles, among whom five patients were punctured four times at L5/S1, and four patients were punctured three times at IJ4/5. The operation time of navigation group was shorter than that of traditional group, and both groups had significant difference (p=0.011). Both groups had no severe complications. There was only one patient with postoperative intervertebral disc residual in the navigation group, which had no direct relation with navigation technology applied in puncture locating. The occurrence rate of complications in both groups had no significant difference (p=0.313, Fig.1-3).

## DISCUSSION

Lumbar intervertebral disc herniation is a common spinal disease. The principle of operative treatment is to relieve mechanical compression of the roots or the cord while guarantying spine integrity and mechanical stability to the greatest degree^4^,^5^ Open operation often requires stripping many muscles beside lumbar vertebra, cutting ligamentum flavum open, destroying vertebral plate and zygapophyseal joint, pulling nerve root and dural sac and causing damage to bony structure of centrum. These operative techniques may adversely affect the stability of the spine. In addition, nerve root and dural sac adhesion as well as other complications may happen easily. With the rapid development of minimally invasive technology, treatment of lumbar intervertebral disc herniation with minimally invasive surgery has become a trend of spine surgery. At present, TESSYS (transforaminal endoscopic spine system) technology proposed by He S et al. on the basis of YESS is frequently used.^6^ TESSYS technology can be used to directly extirpate the protruding nucleus pulposus through intervertebral foramen, and directly reduce pressure of nerve root, which neither damages the muscle at the rear of lumbar vertebra nor damages the structure of important arthrosis ligament of lumbar vertebra. Without the obvious impact on lumbar vertebra stability, it has the advantages of small operative wound, short time in bed, low operation cost and fast recovery. Therefore, TESSYS technology has the better curative effect and the wider indication. Currently, it has been accepted by clinicians and patients.^7^ Puncture locating is the core content of TESSYS technology. However, percutaneous intervertebral foramen microscope technology learning curve is steep, and puncture locating accuracy is high. Due to the reasons such as narrow puncture safety space, anatomical structure sheltering and poor 3D sense and immature operation skills of beginners, puncture locating difficulty, extended operation time, increase of patients’ puncture pains, increase of dural sac and nerve root damage risk and increase of X-ray contact caused by repeated fluoroscopy are often caused.^8^,^9^ Spine surgeons should carefully consider how to reduce puncture and fluoroscopy times, shorten operation time, and improve operation safety and enhance the curative effect of disk removal . Thus, advent of 3D guiding device for puncture reduces radiation and improves puncture accuracy rate. Moreover, intervertebral foramen microscope locator was designed to assist puncture locating, and the good puncture effect was gained. But, the learning and application process is still complex, so it is hard to popularize clinically.^10^ Since early 1990s, computer navigation technology has begun to be applied in spinal surgery. It has been frequently reported that that computer navigation can promote operation accuracy.

The common risks of intervertebral foramen technology mainly include intervertebral space infection, nerve root damage, endorhachis avulsion, hemorrhage, intestinal tract and blood vessel damage, etc.^11^ It merits a discussion as to how to reduce surgical complications and improve operation treatment. Some researchers have reported that the occurrence of complications after intervertebral foramen microscopic operation was 4.89%.^12^,^13^ If puncture locating is not accurate enough, blind puncture may result in dural sac rupture, poor work channel position, difficulty in removing nucleus pulposus tissue, and the inability to reduce pressure thoroughly and relieve waist and leg pains after the operation. Even, the damage may be attached to protruding nucleus pulposus tissue or never root on the fiber ring. In this study, the navigation group had no complication. Postoperative follow-up visit of lumbar vertebra MRI promoted that, protruding nucleus pulposus tissue could be excised completely. In the traditional group, nerve root pain was reported by one patient in the puncturing process. After 1-week of conservative treatment, the symptom disappeared. Non-dynamic location of puncture might damage the nerve root. Postoperative lumbar vertebra MRI of two patients promoted that, nucleus pulposus tissue excision was insufficient. This may be because target spot of intra-operative puncture was not the ideal position, and the work channel of intervertebral foramen microscope was not placed well enough. This study indicates that accurate puncture and target locating contribute to direct extirpation of intervertebral disc under the microscope. Target puncture is the key to the success of intervertebral foramen microscopic operation.^14^,^15^ In clinical practice, we have experienced that MR technology can achieve target puncture based on computer navigation, and its advantages are as follows: (1) Preoperative computer navigation can dynamically adjust puncture direction and position in the puncturing process, and improve the success rate of reaching the target spot through one puncture;^16^,^17^ (2) it can assist the doctors lacking percutaneous intervertebral foramen microscopic experience in improving puncture level, reducing puncture and fluoroscopy times, and shortening operation time; (3) it can shorten learning cycle of young doctors learning intervertebral foramen microscopic technology and enhance operators’ confidence.

Clinically, puncture is required for many deep tissues and organs for diagnosis or treatment, such as intracranial puncture and drainage, tumor biopsy and nephrolithotripsy. The puncture target spot is often in the deep of body surface. The proper needling angle and depth are the key to puncture success.^18^,^19^ Meanwhile, how to avoid by-damage in the puncture channel is also the key to reducing puncture times and complications. In practical operation, internal puncture target spot is sheltered by surface skin, muscle and other tissues. The important anatomical structures often exist in the puncture path, which brings about great challenges to puncture operators. MR technology presents the overlaying effect of virtual images and real images through pad and mobile phone when the computer processes patients’ image data in advance, i.e. projecting punctured organs transparently on the body surface is technically feasible.

### Authors’ Contributions:

**MG &** **Hong-xun Cui** designed this study and prepared this manuscript, and are responsible and accountable for the accuracy of the work.

**JW:** Collected and analyzed clinical data.

**SY:** Significantly revised this manuscript.
